# Biochar As Plant Growth Promoter: Better Off Alone or Mixed with Organic Amendments?

**DOI:** 10.3389/fpls.2017.01570

**Published:** 2017-09-15

**Authors:** Giuliano Bonanomi, Francesca Ippolito, Gaspare Cesarano, Bruno Nanni, Nadia Lombardi, Angelo Rita, Antonio Saracino, Felice Scala

**Affiliations:** ^1^Dipartimento di Agraria, University of Naples Federico II Portici, Italy; ^2^School of Agricultural, Forestry, Food and Environmental Science, University of Basilicata Potenza, Italy

**Keywords:** organic amendment, crop productivity, C/N ratio, N content, organic matter quality, “*terra preta*”, ^13^C CPMAS NMR

## Abstract

Biochar is nowadays largely used as a soil amendment and is commercialized worldwide. However, in temperate agro-ecosystems the beneficial effect of biochar on crop productivity is limited, with several studies reporting negative crop responses. In this work, we studied the effect of 10 biochar and 9 not pyrogenic organic amendments (NPOA), using pure and in all possible combinations on lettuce growth (*Lactuca sativa*). Organic materials were characterized by ^13^C-CPMAS NMR spectroscopy and elemental analysis (pH, EC, C, N, C/N and H/C ratios). Pure biochars and NPOAs have variable effects, ranging from inhibition to strong stimulation on lettuce growth. For NPOAs, major inhibitory effects were found with N poor materials characterized by high C/N and H/C ratio. Among pure biochars, instead, those having a low H/C ratio seem to be the best for promoting plant growth. When biochars and organic amendments were mixed, non-additive interactions, either synergistic or antagonistic, were prevalent. However, the mixture effect on plant growth was mainly dependent on the chemical quality of NPOAs, while biochar chemistry played a secondary role. Synergisms were prevalent when N rich and lignin poor materials were mixed with biochar. On the contrary, antagonistic interactions occurred when leaf litter or woody materials were mixed with biochar. Further research is needed to identify the mechanisms behind the observed non-additive effects and to develop biochar-organic amendment combinations that maximize plant productivity in different agricultural systems.

## Introduction

Biochar is a carbon-rich product defined as “a solid material obtained from the thermo-chemical conversion of biomass in an oxygen limited environment” (International Biochar Initiative [IBI], 2012). Biochar is generated through pyrolysis, i.e., a thermal process carried-out at temperatures that range from 250°C to >900°C and under limited oxygen availability. Many positive interplays with soils are related to the biochar including the liming effect (Van Zwieten et al., 2010), the increase of water retention capacity (Novak et al., 2012) and the capability to adsorb phytotoxic organic molecules (Oleszczuk et al., 2012), stimulate the activity of beneficial microbes (Warnock et al., 2007) and suppress soilborne pathogens (Bonanomi et al., 2015). The beneficial effects of biochar on crops production have been known since ancient times. In Amazon basin, pre-Columbian populations developed the “*terra preta*” soils, also known as “Amazonian dark earth,” by repeating cycles of vegetation burning combined with the application of organic amendments including leaf litter, nutrient-rich kitchen wastes, and fecal materials (Kammann et al., 2016). However, scientific evidence in support of such ancient agricultural practice has taken place only in the last two decades (Lehmann and Joseph, 2015). For these reasons, biochar is nowadays largely used as a soil amendment and commercialized worldwide (Jirka and Tomlinson, 2015).

The meta-analyses of Jeffery et al. (2011) and Biederman and Harpole (2013), however, highlight that the beneficial effect of biochar on crop productivity is context dependent, with several studies reporting negative crop responses (Wisnubroto et al., 2010; Calderón et al., 2015; Knox et al., 2015). In this regards, the combined use of biochar with other, non-pyrolyzed organic amendments (i.e., compost, manure, plant litter, etc.) has been proposed, but, to date, only incomplete results are available on this topic. On the other hand, a flourishing number formulations, in which biochar is combined with different types of organic matter to obtain the so called “*terra preta*”-like planting substrates, have been commercialized (e.g., Wolf and Wedig, 2007). The recent review by Kammann et al. (2016) acknowledges that very few studies that directly combined organic amendments with biochars are available. Furthermore, most of the research concerns co-composting, e.g., the technique where biochar is added to compost at the beginning of the process. Most of these studies reported that “activated” biochar by co-composting consistently promotes plant growth, with better performances than those observed when composts and biochars are used individually (Joseph et al., 2013; Schulz et al., 2013). As recently demonstrated, plant growth improvement by co-composted biochar is largely mediated by nutrients capture, especially nitrate, and their subsequent slow release (Kammann et al., 2015).

This body of evidence suggests that mixing biochar with fresh organic matter is a powerful factor to explicate the potential positive effects on higher plants by the application of such organic amendments. However, because of the limited and fragmented knowledge, reliable guidelines about the types and amount of organic materials that should be mixed with biochars to maximize plant growth are lacking. The chemical diversity of biochars, related to the quality of the initial organic feedstock and pyrolysis conditions, together with the large variety of organic matter types used in agriculture (e.g., composts, crop residues, humic substances, peat and organic wastes from agro-industry) define a great number of possible biochar-organic matter combinations. In this context, the general aim of this work was to study the effects of biochars and organic matters alone and in mixture on crop performances. For this purpose, we analyzed the effect of 10 biochars and 9 not pyrogenic organic amendment types (thereafter indicate as NPOA), alone or in all possible mixtures (i.e., 90 combinations), on lettuce growth (*Lactuca sativa* L.). We selected different organic matter types, ranging from compost to leaf litter, in order to cover materials with a wide spectrum of organic carbon chemistry and total nitrogen content. All organic materials were characterized by standard chemical analyses and by ^13^C-cross-polarization magic angle spinning (CPMAS) nuclear magnetic resonance (NMR) spectroscopy (Kögel-Knabner, 2002) to investigate the link between organic matter chemistry and crop responses. Specific aims of the study were to (i) assess the effect of 10 biochars, 9 NPOA types, and their 90 combinations on growth of lettuce; (ii) explore the relationships between organic feedstocks and biochar chemistry, as defined by ^13^C-CPMAS NMR spectroscopy and elemental analysis (pH, EC, C, N, C/N, and H/C ratios), and growth of lettuce; (iii) identify, starting from the initial chemical composition, the biochar/organic matter combinations that maximize plant growth.

## Materials and Methods

### Organic Amendment Collection

Nine NPOAs were selected because represent materials with a wide range of organic carbon chemistry and total nitrogen (N) content: F.O.R.S.U., is the organic fraction of municipal solid waste (Naples municipality, Southern Italy), wood chips from sawmill, *Medicago sativa* L. hay, *Zea mays* L. stalks, a compost from cattle manure, and four freshly fallen leaf litter types (*Quercus ilex* L., *Fagus sylvatica* L., *Robinia pseudoacacia* L., and *Populus alba* L.). In the case of leaf litter, for each species freshly abscised leaves were collected from randomly selected plants (*N* > 10), air dried for 25 days until reaching constant weight, and then was stored at room temperature.

### Biochar Production

Ten biochar types were used in this study: two were purchased and eight produced in our laboratory. The eight biochar types were made from four feedstock (F.O.R.S.U., *Medicago* hay, *Zea* stalks, and wood chips) that were subject to pyrolysis at two temperatures (300 and 550°C) for 5 h in a muffle furnace. Activated carbon (AC) and a biochar from *Fagus* wood were purchased from Sigma–Aldrich Co., and a local market, respectively. All NPOAs and biochars were powdered in a blender to obtain <2 mm particles and then stored in air-tight containers.

### Chemical Analyses and ^13^C-CPMAS NMR Characterization

Electrical conductivity (EC) and pH of the NPOAs and biochars were measured by using a pH-meter (Basic 20 CRISON) and conductivity meter (CRISON) in suspensions of 1:2.5 and 1:5 organic material:water, respectively. NPOAs and biochars were characterized for total H, C, and N content by flash combustion of micro samples (5 mg) using an Elemental Analyser NA 1500 (Fison 1108 Elemental Analyzer, Thermo Fisher Scientific).

All materials were characterized by ^13^C-CPMAS NMR obtained in the solid state under the same conditions to allow a quantitative comparison of spectra. A Bruker AV-300 spectrometer equipped with a 4 mm wide-bore MAS probe was used. NMR spectra were obtained with MAS of 13000 Hz of rotor spin, 1 s of recycle time, 1 ms of contact time, 20 ms of acquisition time, 2000 scans. Samples were packed in 4 mm zirconium rotors with Kel-F caps. The pulse sequence was applied with a 1H ramp to account for non-homogeneity of the Hartmann-Hahn condition at high spin rotor rates. The ^13^C-CPMAS NMR spectrum was automatically integrated to calculate the area of the peaks which appeared in the chosen region. Seven spectral regions were selected as identified by previous studies (Kögel-Knabner, 2002; Bonanomi et al., 2016a): 0–45 ppm = alkyl C; 46–60 ppm = methoxyl and *N*-alkyl C; 61–90 ppm = *O*-alkyl C; 91–110 ppm = di-*O*-alkyl C; 111–140 ppm = H- and C- substituted aromatic C; 141–160 ppm *O*-substituted aromatic C (phenolic and *O*-aryl C); 161–190 ppm carboxyl C.

### Plant Bioassay

A plant bioassay was carried-out to determine the effect of NPOAs and biochars on the growth of lettuce (*Lactuca sativa* L.), a vegetable cultivated worldwide. Seeds of lettuce were purchased at a local market.

The bioassay was carried-out with 9 NPOAs and 10 biochars used pure plus the untreated control. In addition, all possible NPOA-biochar mixtures were made, resulting in 90 organic matter combinations, replicated 10 times with a total of 1,100 experimental units. Each organic amendment was applied in a pot alone at 2% (10 g^-1^ per pot of dry matter), and carefully mixed with the soil; the unamended soil was also mixed in the same manner. The pots of the two-organic matter mixtures (90 combinations) were amended with 2% of dry organic amendment (10 g^-1^ per pot) having a loading ratio of 50:50. The unamended soil was set as the control.

For this bioassay, about 800 kg of a previously characterized fertile soil were collected from the top 20 cm layer in an agricultural field. Soil was collected in a very productive area of about 5,000 ha cultivated under greenhouses located in the Salerno area (Southern Italy). The study site is characterized by a Mediterranean climate with a mean annual temperature of 15.9°C and monthly temperatures ranging from 23.6°C in August to 9.0°C in January. Mean annual rainfall does not exceed 988 mm with a relatively dry summer (84 mm). Low-technology, unheated polyethylene-covered greenhouses (height ∼4 m) are the main crop protection structures used in this area (Bonanomi et al., 2016b). According to Soil Survey Staff classification (Soil Survey Staff, 1998), the soil was Lithic Haplustolls (Supplemenatry Table S1). The soil was sieved in the laboratory (mesh size < 2 mm). Pots (14 cm diameter and 16 cm height) were filled with 500 g of air-dried soil and planted with 1 pre-germinated 7-day-old seedlings. Then, pots were placed in a greenhouse (20 ± 5°C during the day and 14 ± 5°C at night) following a completely random design with regular rotation every 10 days. Pots were wetted with distilled water every 3 days until water holding capacity was reached. After 90 growing days, above-ground plant part were harvested, dried (80°C in a ventilated oven until constant weight was reached), and dry weighted.

### Data Analysis

#### Not Pyrogenic Organic Amendment and Biochar Chemistry

One-way ANOVA was used to assess differences in litter chemistry as evaluated by chemical analyses and ^13^C CPMAS NMR. Spectral data from ^13^C NMR analysis were also explored with a multivariate cluster analysis (CA) in order to provide a synthetic representation of the variability of NPOA and biochar chemistry.

#### Not Pyrogenic Organic Amendment and Biochar Used Pure

Concerning the bioassay results, the species response data were expressed as a percentage of the respective control, then subjected to one-way ANOVA to assess the effect of NPOAs and biochars used pure on the growth of target plant species. Pairwise differences were tested using Duncan *post hoc* test, and statistical significance was set at *P* < 0.05. To analyze the relationship between the target species in the bioassays and NPOA and biochar biochemistry, correlation was extensively calculated between plant growth and ^13^C NMR data, as well as basic chemical parameters (i.e., pH, EC, C and N content, C/N and H/C ratios) for the same test material. Plant growth was also tested for correlation with ^13^C-CPMAS NMR spectral regions (*N* = 7) selected from reference literature (Kögel-Knabner, 2002; Bonanomi et al., 2016a). Finally, ^13^C NMR spectral data were submitted to multivariate principal component analysis (PCA), in order to assess the multiple relationships among NPOM and biochar chemistry with plant growth. Following the approach indicated by Legendre and Legendre (1998) for supplementary variables, bioassay growth was also plotted as a loading vector in the bi-dimensional PCA space even if it was not used to compute the eigenvalues of the same ordination space. Analyses were performed with Statistica 10 (Statsoft, Inc., Tulsa, OK, United States).

#### Not Pyrogenic Organic Amendment and Biochar Used in Mixture

Mixed NPOMs and biochars were analyzed by a traditional method used to specifically evaluate synergism and antagonism of non-additive effects. In this case, lettuce growth observed in the presence of different mixtures have been compared with the values expected from the component NPOA and biochar used pure. This approach resembles that used to address the effect of litter mixing on mass loss during decomposition (Wardle et al., 1997; Bonanomi et al., 2010). For all mixtures, the expected lettuce growth was calculated as described by Salamanca et al. (1998) as follow:

$$E_{LG}=\overset{S}{\underset{i=1}{\Sigma }}O_{LGi}\times P_{LGi}$$

where *E*_LG_ is the expected mass loss (%), *O*_LG_*_i_* is the observed lettuce growth in case of amendment with NPOM or biochar alone, and *P*_LG_*_i_* is the proportion between NPOM and biochar in the mixture. For each NPOA-biochar mixture, paired *t*-test was used to assess significant differences between observed and expected values (Salamanca et al., 1998; Wardle et al., 2006). Classification of NPOA-biochar interactions about lettuce growth in the mixtures followed that used by Gartner and Cardon (2004): additive (no significant differences between observed and expected values), non-additive synergistic (observed higher than expected), non-additive antagonistic (observed lower than expected).

Finally, with the aim of identifying the chemical parameters of the NPOAs and biochars that maximize plant growth when the materials were mixed, a linear regression was performed using the ‘*lm*’ function in R version 3.3.0 (R Core Team, 2016). With this approach, we identified the significance of a set of descriptors of NPOA and biochar chemistry (pH, EC, C and N content, C/N and H/C ratios, spectral regions from ^13^C CPMAS NMR) used as predictors on a response variable (i.e., observed-expected ratio of plant growth). In addition, the quantification of the relative contribution (expressed as percent of explained variance) of each predictor for the response variable was assessed by using the natural decomposition method (*lmg*, Lindeman et al., 1980) as well as bootstrap estimates for the confidence intervals of the metrics, with the package ‘*relaimpo’* (Grömping, 2006).

## Results

### Biochar and Not Pyrogenic Organic Amendment Chemistry

Initial nutrient concentrations and organic C chemical fractions significantly varied between biochars and NPOAs (**Table 1**). The highest N concentration was found in leaf litter from N fixer species (i.e., *Medicago* and *Robinia*), in compost, F.O.R.S.U. and biochars derived from N rich feedstocks (**Table 1**). C/N ratio showed large variations, ranging from the 639 value of wood biochar made at 300°C to the 6.41 value of *Medicago* hay (**Table 1**). EC was high for compost, F.O.R.S.U., *Medicago* hay and biochars derived from these feedstocks (**Table 1**). For NPOAs the pH showed small variations, ranging from 5.35 to 7.10 values of *Fagus* and *Medicago* litter, respectively. Biochar, instead, showed a much larger variation of pH, ranging from 5.67 of AC to 11.60 of *Medicago* biochar made at 550°C (**Table 1**). The H/C ratio of NPOAs was in all case >1.71 with the only exception of compost (1.32). In contrast, pyrolyzation drastically reduced the H/C ratio giving values ranging from 0.21 of AC to 1.11 of *Medicago* biochar made at 550°C (**Table 1**).

**Table 1:** Main characteristics of not pyrogenic organic amendment (NPOA) and biochar assessed by elemental and ^13^C cross-polarization magic angle spinning CPMAS nuclear magnetic resonance (NMR) analyses.

	Elemental and chemical parameters					^13^C-CPMAS NMR-derived parameters						
		
	pH	EC (μS/cm)	*N* (%)	C/N	H/C	Carboxylic C 161–190 ppm	*O*-substituted aromatic C 141–160ppm	H-C_-_substituted aromatic C 111–140 ppm	di-*O*-alkyl C 91–110 ppm	*O*-alkyl C 61–90 ppm	Methoxyl and *N*-alkyl C 46–60 ppm	Alkyl C 0–45 ppm
**NPOA**												
F.O.R.S.U.	6.84d	4950d	4.77b	10.71g	1.71b	8.22ab	1.05f	5.37e	7.74c	40.21b	11.64a	25.57c
*Medicago*	7.10cd	6430c	6.34a	6.41h	2.17a	11.24a	2.04e	6.55e	8.79c	39.27b	9.54b	22.57c
Wood powder	6.68d	152i	0.12	400.04b	2.12a	3.10d	3.90d	8.64d	14.95a	58.07a	5.35c	6.01f
*Zea*	6.54d	2224g	0.70f	57.68d	2.31a	2.63e	1.31f	2.93f	14.24a	62.63a	6.77c	9.39e
*Fagus*	5.35e	1043	2.09d	18.62f	1.98b	6.37b	6.12c	10.73d	12.38a	39.44b	8.13b	16.83d
*Populus*	6.72d	2958ef	1.45e	25.39e	1.78b	6.62b	5.11c	10.60d	12.48a	41.61b	7.06bc	16.51d
*Quercus*	5.23e	1130h	1.14e	28.82e	1.98b	3.91 d	5.71c	10.61d	12.02a	41.87b	8.35b	17.53d
*Robinia*	6.51d	2781 f	2.63d	12.45g	2.27a	8.23ab	5.09c	8.84d	10.48b	35.66c	9.51b	22.19c
Compost	6.52d	13643a	3.13c	13.09g	1.32c	3.97d	2.06e	6.32e	8.59c	34.17c	15.48a	29.41c
**Biochar**												
F.O.R.S.U. 300°C	7.98c	3840e	6.01a	10.01g	1.05d	3.89d	8.65b	28.95c	5.86d	5.50d	5.13c	42.25a
F.O.R.S.U. 550°C	11.41a	10600b	6.26a	9.78g	1.04d	2.87e	12.28a	71.85°	4.45d	2.37d	1.29e	4.48f
*Medicago* 300°C	11.00a	7530c	4.48b	12.15g	1.09d	4.27c	7.55bc	27.15c	4.51d	3.87d	4.94d	47.41a
*Medicago* 550°C	11.60a	9490b	4.43b	11.78g	1.11d	4.36c	8.83b	71.41a	5.57d	4.89d	1.58e	2.93g
Wood 300°C	7.77c	138i	0.13g	639.04a	0.49ef	1.80	13.97a	39.33b	8.14c	5.97d	9.19b	21.69c
Wood 550°C	10.66a	218i	0.21g	410.68b	0.66e	4.24c	5.94c	64.86a	5.43d	5.49d	4.19d	9.39e
*Zea* 300°C	9.13b	2107g	2.48d	27.24e	0.38f	3.23d	11.31a	32.55	7.05c	6.89d	6.30c	32.51b
*Zea* 550°C	10.86a	3480e	2.46d	27.95e	0.74e	2.06e	9.64b	75.66a	4.91d	3.83d	1.57e	2.26g
Activated carbon	5.67e	264i	1.47e	51.70d	0.21g	5.67c	7.13bc	49.10b	15.47a	10.53d	5.03d	7.07e
Commercial biochar	7.49c	290i	0.50f	149.62c	0.37f	7.70b	3.76d	62.00 a	7.76c	5.52d	4.17d	9.10e


*For each parameter, different letters within each column indicate significant differences (Duncan *post hoc* test at *P* < 0.05).*

The ^13^C-CPMAS NMR data of NPOAs and biochars, with regard to C molecular types corresponding to reference spectral regions, revealed consistent changes of the material organic chemistry (**Table 1**). Among NPOAs, ^13^C NMR spectra showed consistent differences of C molecular types, with major chemical changes observed for the alkyl-C fraction (0–45 ppm), significantly lower for *Zea* and wood, and the methoxyl and *N*-alkyl C fraction (46–60 ppm) that was higher in F.O.R.S.U., compost, *Medicago*, and *Robinia* litter (**Table 1**). The relative abundance of the *O*-alkyl-C fraction (61–90 ppm) was higher for wood and *Zea*, and quite low for compost (**Table 1**). The carboxylic C fraction (161–190 ppm) ranged from 11.24 to 2.63% of *Medicago* hay and *Zea* residues, respectively. The H-C-substituted aromatic C region (111–140 ppm) was very high for lignin rich plant litter (i.e., *Fagus*, *Populus*, and *Quercus*) (**Table 1**). Interestingly, the chemistry of biochars was dramatically different from that of NPOAs. In particular, di-*O*-alkyl-C, methoxyl and *N*-alkyl C, and carboxylic C fractions were very low compared to those of NPOAs (**Table 1**). The *O*-alkyl-C region was abundant only in biochars made at 300°C, but disappeared in all biochars made at higher temperatures (**Table 1**). In contrast with the other spectral regions, the aromatic C fractions (141–160 ppm, but especially that at 111–140 ppm) showed a sharp increase in biochars compared to NPOAs, with the highest values recorded for biochar made at temperatures >300°C (**Table 1**).

**FIGURE 1:**
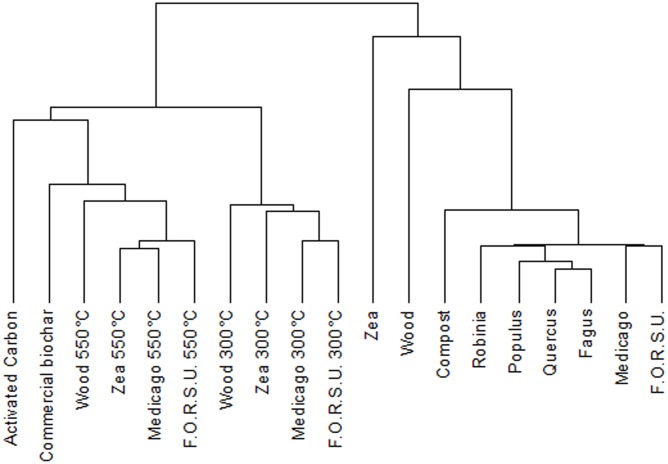
Dendrogram from cluster analysis of the 19 organic materials (10 biochar and 9 not pyrogenic organic amendment).

Cluster analysis of NPOA and biochar spectra showed a remarkable difference between the two groups (**Figure 1**). NPOAs were mostly grouped together at low distance values, with only wood and *Zea* that had major distances, indicating some species-specific chemical differences (**Figure 1**). The dendrogram of biochars based on ^13^C NMR spectra showed two main clusters segregating at high distances and including materials made at either low (300°C) and high-intermediate (550°C, AC and commercial biochar) temperatures (**Figure 1**). Moreover, within the low-temperature cluster, most samples belonging to the same feedstock were consistently aggregated at the lowest distance levels, while within the high-temperature cluster sample aggregation was independent of the feedstock of origin (**Figure 1**).

### Effect of Biochar and Not Pyrogenic Organic Amendment on Plant Growth

**FIGURE 2:**
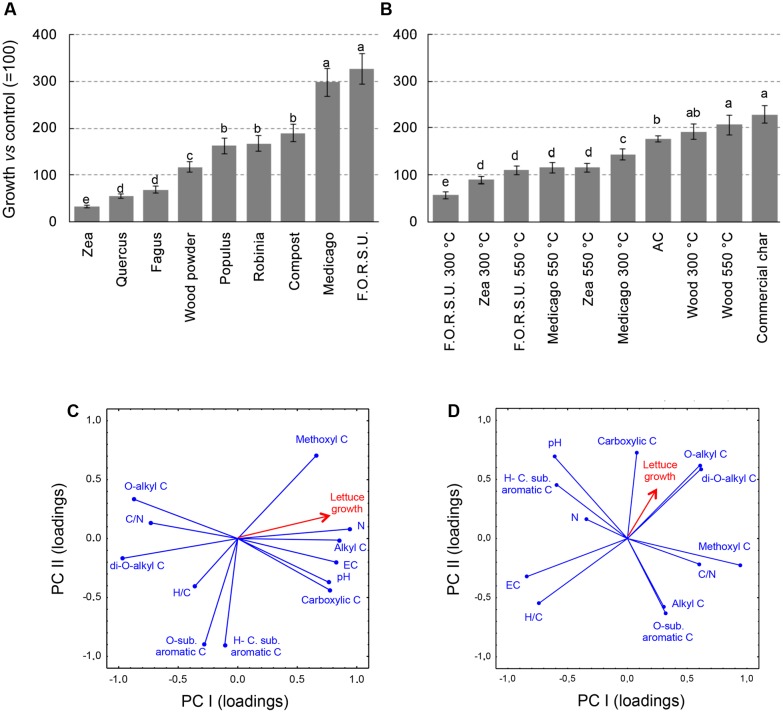
Growth of lettuce (*Lactuca sativa*) plants in pot amended with not pyrogenic organic amendment **(A)** and biochar **(B)** at 2% (w/w). Values are expressed as percentage of untreated controls (=100%). Different letters indicate significant difference with comparison within each organic amendment type (Duncan test at *P* < 0.05). Principal component analysis (PCA) between not pyrogenic organic amendment **(C)** and biochar **(D)**, based on basic chemical parameters and seven ^13^C NMR spectral regions with lettuce growth. Panel also shows target lettuce growth as supplementary variables following Legendre and Legendre (1998).

The effect of NPOAs and biochars used alone on plant growth was largely affected by the type material (**Figures 2A,B**). With NPOAs lettuce showed various responses, being inhibited by *Fagus*, *Quercus*, and *Zea*, not affected by wood powder and promoted by the remaining materials, especially *Medicago* hay and F.O.R.S.U. (**Figure 2A**). Among biochars, five materials stimulated lettuce growth compared to untreated control, three had no significant effects and only two showed a moderate inhibition (i.e., F.O.R.S.U. and *Zea* biochar made at 300°C) (**Figure 2B**).

The PCA results provided a synthetic picture of the chemistry-dependent effects on the target plant responses caused by NPOAs and biochars (**Figures 2C,D**). PCA provided a satisfactory ordination of the NPOA and biochar chemical parameters in relation to the sensitivity of the tested plant, with the eigenvalues of the first two components accounting for 76.53% (53.13 and 23.40%) and 62.47% (35.82 and 26.65%) of the total variance for NPOAs and biochars, respectively. **Figures 2C,D** reports the loading vectors that represent the organic matter chemistry parameters plotted in the bi-dimensional space. For NPOAs, the first and second PCA components highlight the importance of pH, N content, EC, C/N ratio, and several NMR regions to affecting plant growth. Lettuce growth was largely explained by the negative correlations with C/N ratio as well as *O*-alkyl C and di-*O*-alkyl C regions, and positive correlations with EC, N content, and the carboxylic C, methoxyl C, and alkyl C NMR regions (**Figure 2C**). For biochars, instead, lettuce growth was explained to its negative correlations with EC and H/C ratio and positive correlations with *O*-alkyl C, di-*O*-alkyl, and carboxylic C NMR regions (**Figure 2D**).

### Synergism, Antagonism, and Additive Effects in NPOA-Biochar Mixtures

**FIGURE 3:**
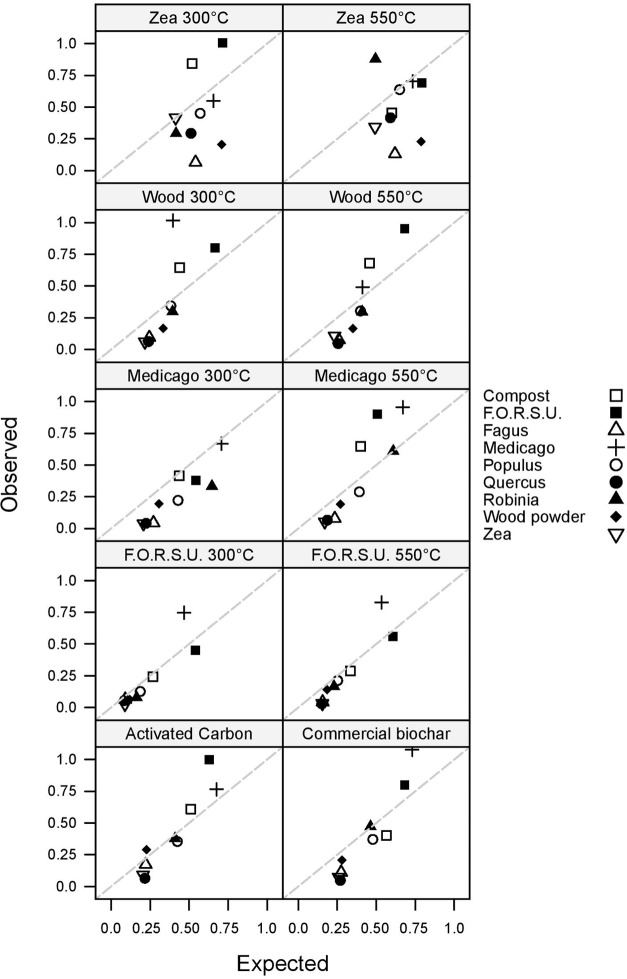
Observed vs. expected values of lettuce growth in mixtures composed of not pyrogenic organic amendment and biochar. Values above and below the 1:1 dashed line indicate synergistic and antagonistic interactions, respectively.

Non-additive and additive effects on plant growth were found in 87.78 and 12.22% of the tested mixtures, respectively (**Figure 3**). Antagonistic responses were more common than synergistic interactions, with 59 and 20 cases, respectively. The magnitude of synergistic and antagonistic interactions ranged from -87.99% to +156.51% of the expected plant growth (**Figure 3**).

**FIGURE 4:**
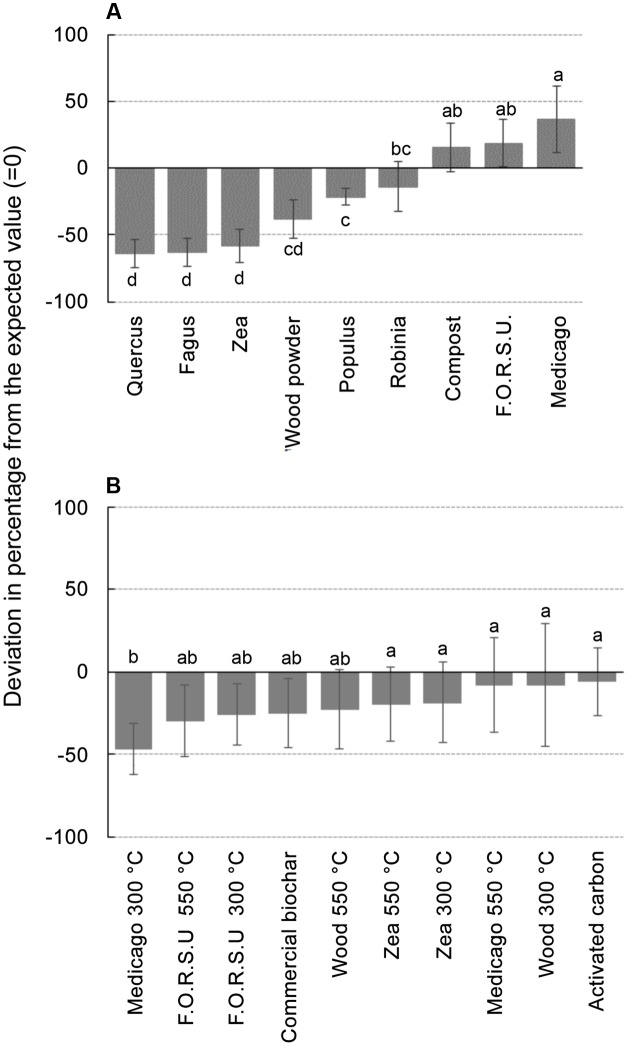
Deviation between observed growths of lettuce in different mixtures compared with the calculated values expected from the growth observed with not pyrogenic organic amendment (NPOA) and biochar used in isolation. In **(A)** vales are the average of 10 biochar combined with each NPOA type, in **(B)** values are the average of 9 NPOA combined with each biochar type. Different letters indicate significant differences (Duncan test at *P* < 0.05).

Biochar and NPOA types in the mixture affected the outcome of non-additive interactions. For instance, when *Medicago* hay and F.O.R.S.U. were used as NPOA, synergistic interactions with biochars were recorded in 7 and 6 out of 10 cases, respectively (**Figure 3**). The deviation between observed and expected growth among all biochar types was +36.74%, +18.72%, and +15.74%, for *Medicago*, F.O.R.S.U. and compost, respectively (**Figure 4**). Mixtures with *Populus* and *Robinia* litter showed synergistic, non-additive, and antagonistic interactions, with the latter being more common than the other two (**Figure 3**). In contrast, the mixtures containing *Quercus* litter as NPOA gave an antagonistic interaction in 90% of the cases, with no synergistic responses (**Figure 3**). Similar results were found for *Fagus* and *Zea* litter as well as with wood which showed 80% of antagonistic interactions and 20% of non-additive responses (**Figure 3**). The deviation between observed and expected growth with all biochar types was negative for *Robinia* and wood powder (**Figure 4**). For *Quercus*, *Fagus*, and *Zea* litter, the deviation between observed and expected growth was -64.05%, -63.15%, and -58.42%, respectively (**Figure 4**).

With biochars, the interactions were additive, i.e., the deviation from expected values was not significant, for AC, *Zea*, and wood biochars made at 300 and 550°C and for *Medicago* char made at 550°C. For the remaining biochars (commercial, *Medicago* 300°C, and F.O.R.S.U. 300 and 550°C), the deviation from expected values was negative with all NPOA types, meaning that antagonist interactions were more important (**Figure 4**).

### Predicting Non-additive Effects from Not Pyrogenic Organic Amendment and Biochar Chemistry

**FIGURE 5:**
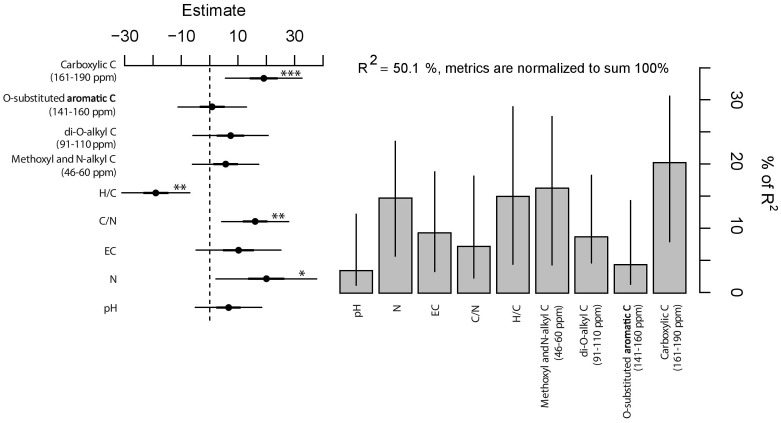
Estimates (filled circles) and 95% confidence interval (bars) of linear regression (left panel). ^∗∗∗^, ^∗∗^, and ^∗^ indicate significance for *P* < 0.001, *P* < 0.01, and *P* < 0.05, respectively. Right panel shows the relative importance (*y*-axis, percentage of variance explained) of predictors with the bootstrapped 95% confidence interval (bars).

Deviation from expected values are adequately described by the linear model [F_(9,80)_ = 8.92, *P* < 0.001, **Figure 5**]; half of the total variance in observed-expected ratio of plant growth is explained by the chemistry of the mixtures. In particular, the significance of individual coefficients indicated a positive influence of carboxylic C (161–190 ppm), C/N ratio, and N content (for *P* < 0.001, *P* < 0.01, and *P* < 0.05, respectively) and a negative effect of H/C ratio (for *P* < 0.01) on the observed-expected ratio of growth. On the other hand, EC and the other C-types corresponding to NMR spectral regions did not reveal statistical significance. Moreover, the relative explained variance vary drastically among chemical parameters, i.e., between 3.5 and 20.3% (**Figure 5**). In this regard, it is noteworthy that the significant variables that contributed the most (20.3% of the relative explained variance) were the carboxylic C (161–190 ppm) followed by the H/C ratio (15.0%), N content (14.8%), and C/N ratio (7.2%). The residual portion of the relative variance is attributed to the non-significant variables.

## Discussion

### Pure Biochar and Not Pyrogenic Organic Amendment

Biochars and NPOAs used pure have contrasting effects on the growth of lettuce, showing clear relationships with their chemical characteristics. Effects of NPOAs on lettuce growth range from intense inhibition (-67% by *Zea* residues) to strong stimulation (+226% by F.O.R.S.U.). The major inhibitory effect was found with N poor materials characterized by high C/N and H/C ratios. Our finding of a severe plant root inhibition by undecomposed litter of *Fagus*, *Quercus*, and *Zea* is consistent with previous studies carried-out in natural ecosystems (Dorrepaal et al., 2007; Lopez-Iglesias et al., 2014; Meiners, 2014). Lettuce growth inhibition by undecomposed leaf litter can partially be attributed to microbial N immobilization (Hodge et al., 2000) and/or to the phytotoxic activity of putative allelopathic compounds (Bonanomi et al., 2011). The N immobilization hypothesis sustains that when organic matter with high C/N ratio is incorporated in soil reduce N availability, thus impairing root system development. The main mechanism is related to the behavior of saprophytic microorganisms in soil. In fact, to efficiently exploit soil organic matter microbes require both organic C and N in a relatively constant stoichiometric ratio. Organic C or N limit microbial growth, and when C/N ratio of organic matter is above the threshold value of ∼30–35 mineral N is taken from the soil, incorporated in microbial biomass and temporarily immobilized (Hodge et al., 2000). Such a hypothesis is indirectly supported by the present work, because plant growth is inversely related to substrate C/N ratio that, in turn, is directly correlated to N immobilization (Palm et al., 2001). However, C/N ratio can only partially explain the observed inhibitory effect because the wood, characterized by the highest C/N ratio, had a neutral effect on plant growth. This discrepancy could be explained by the quality of organic C of the NPOA. We found, in fact, that plant growth was inversely related to the relative abundance of the *O*-alkyl and di-*O*-alkyl NMR regions that are mainly associated with sugar and polysaccharides (Kögel-Knabner, 2002). This result suggests that organic amendments rich in labile C, e.g., sugars and relatively simply polysaccharides, may induce a faster N immobilization with a more negative impact on crop growth than materials rich in lignin (e.g., powdered wood). An alternative hypothesis is that plant litter can release, either directly or mediated by microbial activity, allelopathic compounds with harmful effects on root growth (Bonanomi et al., 2011). The identification of the ultimate causes of litter inhibitory is out of the aim of this work, but our approach by using ^13^C NMR indicates that lettuce growth is negatively affected by *O*-alkyl and di-*O*-alkyl C, but positively associated with signals related to plant tissue lignification. On the other hand, we found that lettuce growth was promoted by NPOAs with a relatively high content of methoxyl C and carboxyl C NMR regions, as well as a high N content. These results confirm the well-known positive effect of mineral and organic N sources on plant development (Marschner, 2012).

Concerning biochars, the effect on lettuce growth was less variable than that observed with NPOAs. Lettuce response ranged from moderate inhibition with F.O.R.S.U. biochar made at 300°C, to a highly significant stimulation observed with AC and the three biochars derived from wood. ^13^C NMR spectra showed that during pyrolysis all materials are subjected to a decrease of labile C molecular compounds, especially at high temperatures, and a relative increase of aromatic fractions. Noteworthy, all biochars pyrolyzed at 300°C showed a peak in the alkyl fraction, mainly related to aliphatic compounds, including the so-called bio-oils (Amonette and Joseph, 2009). However, the alkyl C region was absent in biochar made at 550°C or higher temperatures (e.g., AC and commercial wood biochar). Biochars made at high temperature or derived from wood sources showed a quite low H/C atomic ratio. This index, which has been proposed to define biochar quality in the context of bioremediation (Xiao et al., 2016), seems to be the best parameter to identify the biochar with the ability to promote plant growth. Finally, it is notable that total N content of biochars (ranging from 0.13 to 6.26% of wood 300°C and F.O.R.S.U. 550°C, respectively) had a limited power to explain its effect on lettuce growth. Probably, during pyrolysis most of the organic N was gradually transformed into pyridine-like compounds, with a consequent drastic reduction of its bio-availability for plants (Bagreev et al., 2001; Chan and Xu, 2009).

### Not Pyrogenic Organic Amendment-Biochar Mixtures

In our experiments non-additive interactions, either synergistic or antagonistic, were prevalent. However, the majority of these interactions showed antagonistic effects, i.e., plant growth was lower than that expected by using each organic amendment alone. When biochars were mixed with N rich materials (i.e., *Medicago* hay, compost or F.O.R.S.U.), synergistic interactions were prevalent.

According to the recent review of Kammann et al. (2016), co-composted biochars have a remarkable plant growth-promoting effect compared to biochars used pure, but no-systematic studies were done to understand the interactive effects of biochars with NPOAs. In our experiment, additive effects were rare considering the expected vs. observed pooled data (11 cases out of 90 combinations). Contrarily to the general trends reported in the literature (Kammann et al., 2016), antagonistic interactions were more common than synergistic ones. However, some important methodological differences between the present and previous studies should be considered. In this study, the organic substrates mixed with biochars span an ample range of N content and C chemistry, while previous studies used only compost (Joseph et al., 2013; Schulz et al., 2013), or selected nutrient-rich materials such as urine (Schmidt et al., 2015).

Our results, being based on a wide range of NPOA types, provided a complete picture to understand the complex relationships between biochar and organic amendments. First, our data highlight that NPOA identity is more important than biochar types as a determinant of plant growth in the mixture. In fact, the deviation from expected values ranged from -29 to -5% for biochar, while for NPOAs the deviation spans from -64 to +36%. This means that most biochars appear suitable as a component of the mixture, with the exception of biochar made from F.O.R.S.U. and *Medicago* at 300°C. The same is not true for NPOAs, because only some materials are able to synergize when are used in combination with biochars. Synergistic interactions were observed with N rich, lignin poor substrates (i.e., compost, F.O.R.S.U., and *Medicago* hay). This result is in agreement with previous findings concerning biochar co-composting, where the best synergism was achieved blending C rich biochars with N rich organic materials (Kammann et al., 2016). The mechanisms proposed to explain the synergism between biochars and compost is based on the improvement of soil structure and water availability (Novak et al., 2012), the promotion of beneficial microbes (Warnock et al., 2007), and the enhanced plant-available nutrients (Liu et al., 2012). A recent study (Kammann et al., 2015) showed that co-composted biochars became highly enriched with nutrients (i.e., nitrate, ammonium, and phosphate) and dissolved organic carbon that is slowly released in soil. In addition to nutrients, low- and high-molecular weight organic C compounds can accumulate on biochar surfaces and in pores, having the potential to affect plant growth. In fact, many low-molecular weight organic C compounds are known to be phytotoxic at high concentrations, but have hormonal effects at low concentrations (Joseph et al., 2013). In this regards, *Medicago sativa* hay and F.O.R.S.U. could be phytotoxic if applied at high rate (Chefetz et al., 1996; Chung et al., 2000), but in presence of biochars such a negative effect could be reversed. Our study, however, was not designed to identify the mechanisms behind antagonistic and synergistic interactions between NPOAs and biochars. In the soil-biochar-NPOA mixtures, all the aforementioned cited mechanisms could be operative, but further studies are needed to clarify their relative contribution.

This study is the first reporting antagonist interactions between NPOAs and biochars. The occurrences of antagonistic interactions are well predictable based on the chemistry of the original components. Antagonisms were observed with wood powder and all leaf litter types, materials which are characterized by low N content combined with a high lignin content and high H/C ratio. Noteworthy, the combination among biochars with *Robinia* leaf litter showed an intermediate behavior, with several additive interactions and some moderate antagonistic interactions. This leaf litter is the only material characterized by a high N concentration and a relatively high lignin content. These results clearly indicate that the combination of biochars with low quality litter types (i.e., high lignin, high H/C ratio, and low N content) would enhance the probability of antagonistic interactions on plant growth. The mechanisms underlying such antagonist interactions are unknown, although a short-term, intense N immobilization could be hypothesized.

## Conclusion

This is the first study that systematically explored the interaction between biochars and organic amendments mixed in the soil and demonstrates that non-additive interactions, either synergistic or antagonistic, were prevalent. The results provided evidence that, at least in the short-term, chemical quality of NPOAs is more important than biochar characteristics in affecting plant responses. In addition, we found that the final effect of the mixtures can be predictable based on the chemical characteristic of their components, with synergistic interactions being prevalent when N rich, lignin poor materials are mixed with biochars. On the contrary, antagonistic interactions occurred when leaf litter or woody materials were mixed with biochars. We acknowledge the limitations of this study which was carried-out by using only one soil type, one target species, and one single loading ratio. Although the selected NPOAs represent a wide range of organic matter chemistry and nitrogen content, several common organic amendments like peat, fresh manure, and humus-like compounds were not included. Therefore, understanding the interactions between biochar and the above-mentioned organic amendment should not be overlooked, with special attention to humic substances that promote root proliferation and growth (García et al., 2016).

Mixing NPOAs with biochar have great potential for plant growth promotion, but their variable performances may be a limiting factor for their extensive use in agriculture. It appears that the positive properties of specific NPOA-biochar combinations far outweigh the growth promotion observed when their component were used separately. However, as long as the impact of NPOA-biochar blending on plant growth remains unpredictable, farmers may be justified in ignoring it as a profitable agronomic technique. In this regard, among other challenges, (i) developing practice guidelines that to ensure reliable biochar mixture performance on plant productivity in different agricultural systems and, (ii) get insight in the chemical and microbiological nature at the base of the synergistic as well the antagonistic interactions among biochar, not-pyrogenic organic matters and soil are strong efforts that researchers will face forward.

## Author Contributions

GB conceived the project. FI, GC, BN, and NL collected litter samples, produced the biochar, performed the experiments, and make chemical analyses. AR, GB, and AS analyzed the data. The manuscript was written by GB, FS, and AS with some contributions from all the other authors. All authors read and approved the final version of the manuscript.

## Conflict of Interest Statement

The authors declare that the research was conducted in the absence of any commercial or financial relationships that could be construed as a potential conflict of interest.
