# Supplementary material for: Biochar As Plant Growth Promoter: Better Off Alone or Mixed with Organic Amendments?
Source: Front Plant Sci. 2017 Sep 15;8:1570. doi: 10.3389/fpls.2017.01570 (PMC5605631; doi:10.3389/fpls.2017.01570)
Supplement: Supplementary file 1 [file Table_1.DOC]

**Table S1.** Chemical and physical properties of the soil used in this study.

| **Parameters** |  | **Value** |
| --- | --- | --- |
| pH |  | 7.74 |
| EC | dS m^-1^ | 0.32 |
| Total CaCO_3_ | g Kg^-1^ | 7.16 |
| Organic carbon | g Kg^-1^ | 15.4 |
| Organic matter | g Kg^-1^ | 26.5 |
| Total N | g Kg^-1^ | 1.60 |
| C/N |  | 9.60 |
| Available phosphorus (P_2_O_5_) | mg Kg^-1^ | 239 |
| Cation exchange capacity | meq 100 g^-1^ | 36.3 |
| Exchangeable potassium | meq 100 g^-1^ | 1.81 |
| Exchangeable magnesium | meq 100 g^-1^ | 6.55 |
| Exchangeable calcium | meq 100 g^-1^ | 27.03 |
| Exchangeable sodium | meq 100 g^-1^ | 0.94 |
